# Metasurface-Coated Liquid Microlens for Super Resolution Imaging

**DOI:** 10.3390/mi16010025

**Published:** 2024-12-27

**Authors:** Tongkai Gu, Kang Wang, Anjiang Cai, Fan Wu, Yasheng Chang, Haiyan Zhao, Lanlan Wang

**Affiliations:** 1 School of Mechanical and Electrical Engineering, Xi’an University of Architecture and Technology, Xi’an 710055, China; tkgu2023@xauat.edu.cn (T.G.); wk1692618205@163.com (K.W.); 2 State Key Laboratory for Manufacturing System Engineering, Xi’an Jiaotong University, Xi’an 710054, China; 3 School of Textile Science and Engineering, Xi’an Polytechnic University, Xi’an 710699, China; fan.wu@xpu.edu.cn; 4 School of Optical and Electronic Information, Suzhou City University, Suzhou 215104, China; cocys@126.com; 5 School of Architecture and Design, Kunshan Dengyun College of Science and Technology, Suzhou 215300, China; zhy_320@126.com

**Keywords:** liquid microlens, super resolution, metasurface, SPR, PNJs

## Abstract

Inspired by metasurfaces’ control over light fields, this study created a liquid microlens coated with a layer of Au@TiO_2_, Core-Shell nanospheres. Utilizing the surface plasmon resonance (SPR) effect of Au@TiO_2_, Core-Shell nanospheres, and the formation of photonic nanojets (PNJs), this study aimed to extend the imaging system’s cutoff frequency, improve microlens focusing, enhance the capture capability of evanescent waves, and utilize nanospheres to improve the conversion of evanescent waves into propagating waves, thus boosting the liquid microlens’s super-resolution capabilities. The finite difference time domain (FDTD) method analyzed the impact of parameters including nanosphere size, microlens sample contact width, and droplet’s initial contact angle on super-resolution imaging. The results indicate that the full width at half maximum (FWHM) of the field distribution produced by the uncoated microlens is 1.083 times that of the field distribution produced by the Au@TiO_2_, Core-Shell nanospheres coated microlens. As the nanosphere radius, droplet contact angle, and droplet base diameter increased, the microlens’s light intensity correspondingly increased. These findings confirm that metasurface coating enhances the super-resolution capabilities of the microlens.

## 1. Introduction

In recent years, the rapid progress in micro- and nanofabrication technologies, coupled with deeper explorations of the microscopic world in life sciences, has led to a growing demand for super-resolution imaging in microscopy systems [1,2]. Researchers have developed innovative microscopic imaging techniques to achieve super-resolution of sub-wavelength nanostructures and overcome diffraction limits in both far-field and near-field regions through ongoing exploration and research. Stimulated emission depletion (STED) microscopy enhances microscopic resolution by modulating the depletion beam’s power, reducing or avoiding far-field transmission wave diffraction [3]. However, STED faces issues like photobleaching of fluorescent probes and phototoxicity in biological samples, limiting its use in live samples. Structure illumination microscopy (SIM) uses the moiré fringe effect to encode high spatial frequency information into lower frequencies, enabling imaging beyond the diffraction limit [4]. However, SIM’s strict light field requirements, susceptibility to reconstruction artifacts, and low contrast restrict its broader application. Stochastic optical reconstruction microscopy (STORM) uses the photoactivation or photoswitching of special fluorescent dyes for single-molecule localization, overcoming the diffraction limit for high-resolution imaging [5]. However, STORM’s slow imaging speed makes it unsuitable for real-time imaging of dynamic processes. Minimal photon fluxes (MINFLUX) technology, using single-molecule localization and quantum photonics, precisely controls fluorescent molecule excitation to surpass the diffraction limit for high-resolution imaging [6]. However, MINFLUX technology is hindered by low imaging throughput and high sample production costs. In contrast, microlenses are compatible with standard microscopes, offer real-time control, have minimal environmental constraints, and ensure high imaging quality [7]. Consequently, employing microlenses to capture evanescent waves [8,9,10,11,12], which are generated from the interaction of light waves with objects and carry high-frequency information while exhibiting high field intensity near the object’s surface that decays rapidly, has emerged as an effective approach to aid microscopes in achieving super-resolution detection [13,14,15,16,17,18,19].

Microlens-assisted microscope technology, though capable of surpassing the optical diffraction limit for super-resolution imaging, encounters substantial challenges when observing structures smaller than 50 nm [20,21]. The primary reasons are the limitations of the space bandwidth product and the discrete sampling capability of detectors [22,23]. The sampling limit, influenced by the number of detector pixels, cannot be increased without a limit. Increasing the pixel count will inevitably raise the detector’s manufacturing costs. The spatial bandwidth product’s limitation on imaging resolution stems from the system’s limited aperture. Thus, the conventional approach to enhancing imaging resolution largely depends on Lukos’ super-resolution concept, which amalgamates image data from multiple small-aperture optical systems to achieve the imaging capabilities of a large aperture system [24,25,26,27]. However, synthetic aperture technology encounters issues like the challenge of capturing high-frequency spectral information and the tradeoff between imaging distance and signal-to-noise ratio, impeding further resolution enhancements.

Nanostructures exhibiting surface plasmon resonance (SPR) effects can concentrate electromagnetic fields in localized areas and amplify them, significantly benefiting signal-to-noise ratio enhancement and offering a novel approach to improving imaging resolution [28,29,30,31,32,33,34,35]. Metasurfaces, composed of these periodic nanostructures, can precisely manipulate the phase, amplitude, and polarization of light waves, effectively modulating the imaging system’s optical transfer function, extending the cutoff frequency, and thus shrinking the spatial domain’s point spread function, achieving angstrom-level single molecule detection sensitivity and spatial resolution. Pengcheng Zhang has developed a microlens-assisted imaging technique that captures the scattering light from plasmonic nanoparticles at the single-particle level, enabling high temporal resolution imaging (up to 5000 Hz) of individual plasmonic nanoparticles. However, the technique faces challenges due to stringent preparation requirements and poor stability [36]. Minkyu Kim’s research demonstrates that nanogap morphology significantly influences plasmon coupling, particularly as increased gap curvature results in reduced coupling energy [37].

Compared to other plasmonic structures, Gold-coated Titanium Dioxide (Au@TiO_2_) Core-Shell nanospheres boast a high refractive index, low loss, and excellent stability, holding promise for metasurface devices in the visible spectrum. Consequently, a liquid microlens coated with a metasurface featuring a periodic arrangement of Au@TiO_2_ Core-Shell nanospheres can enhance microlens imaging resolution through plasmonic effects. It is described how to fabricate a metasurface-coated liquid microlens using the lift-off method and liquid self-assembly technology, as follows. First, prepare the base with circular holes for the microlens array through traditional photolithography and subsequent photoresist reflow processes. Then, immerse the base in a solution containing nanospheres until it is completely submerged. Subsequently, at the gas–liquid–solid triple phase interface, under the combined action of interfacial forces and shear stress, the liquid self-assembles on the base to form a specific functional shape. By vertically lifting the base, the nanospheres automatically stay on the surface of the base, maintaining a closely packed specific functional shape [38,39]. However, the requirements of the experiment are difficult to achieve. Given that plasmon excitation involves electron oscillation, it can cause significant local Joule heating [40,41], and nanospheres tend to aggregate, couple, and melt at high temperatures under high power, adversely impacting the super-resolution imaging results and practical applications. However, the liquid’s good thermal conductivity can dissipate the Joule heat generated by the plasmon, mitigating its impact on super-resolution imaging results. This approach is crucial for further advancing the super-resolution imaging capabilities of microlens-assisted microscopes.

In this paper, we designed a compound structure, which is the uniform coating of Au@TiO_2_, Core-Shell nanospheres on the surface of a liquid microlens. Utilizing the plasmonic effect of Au@TiO_2_, Core-Shell nanospheres, we extended the imaging system’s cutoff frequency, thereby enhancing the liquid microlens’s super-resolution capabilities. We investigated the super-resolution mechanism of the metasurface-coated liquid microlens using the finite difference time domain (FDTD) method. Optimizing parameters such as the sample contact width, nanosphere size, and droplet’s initial contact angle significantly improved super-resolution imaging performance. The results indicate that, under plane wave illumination, the light intensity of the Au@TiO_2_, Core-Shell coated microlens (5 nm radius) increased, and the uncoated microlens had an FWHM of 1.69 µm, whereas the Au@TiO_2_, Core-Shell nanospheres coated microlens had a FWHM of 1.56 µm, which is 1.083 times larger when compared to the Au@TiO_2_, Core-Shell-coated microlens, demonstrating a significant advantage in super-resolution imaging. This innovative detection technology holds significant potential for applications in biomedical imaging, environmental monitoring, and industrial testing.

## 2. Materials and Methods

Surface plasmons are electromagnetic oscillations occurring at the interface between conductors and dielectric materials. Essentially, this oscillation represents the intense resonant response of electrons to external excitation light. Surface plasmons, having a shorter wavelength than the excitation light, enable denser interference patterns, leading to a greater optical frequency shift in the spectral domain and, consequently, higher spatial resolution in the final reconstructed image.

In the transverse magnetic mode, the dispersion relation of surface plasmon polaritons (SPRs) is determined by solving Maxwell’s equations in conjunction with the boundary value conditions of the electromagnetic field, as detailed below:

(1) $$k_{spr}=k_{0}\sqrt{\frac{\varepsilon_{m}\varepsilon_{d}}{\varepsilon_{m}+\varepsilon_{d}}}$$

where ***k*_0_** *= w/c* is the wave number in a vacuum. Therefore, SPR can only occur when ***k*_0_** is consistent with the wave number of the incident light wave. $\varepsilon_{d}$ and $\varepsilon_{m}$ represent the refractive index of dielectric material and metal, respectively. The dielectric constant of metal materials is a complex number, which means that when the incident light passes through the dielectric material and is incident on the metal surface, the wave number of SPR is greater than that of the dielectric material. This is shown below.

(2) $$\left|k_{spr}\right|>\left|k_{0}\sqrt{\varepsilon_{d}}\right|$$

Based on Fourier optics, the optical transfer function (OTF) of an imaging system corresponds to the Fourier transform of its point spread function (PSF). The PSF of the system cannot be in the form of the δ function but has a certain width, so the OTF of the system cannot occupy an infinite area in Fourier space but can only occupy a limited range. The imaging system captures high-frequency component information from the observed object that is below a specific cutoff frequency. The magnitude of the cutoff frequency dictates the imaging system’s resolution. Consequently, enhancing the imaging system’s cutoff frequency or narrowing its PSF are effective methods for improving microscopic imaging resolution.

Achieving PSF compression and increasing the cutoff frequency are typically challenging in most systems. Fortunately, spectrum shifting technology allows us to move high-frequency components of the observed object, which are outside the cutoff frequency, into the area within the imaging system’s OTF cutoff frequency without altering the OTF cutoff frequency, thus enhancing the imaging resolution. Assuming the translation amount is $\vec{k}$, the OTF passband of the system after translation can be expressed as follows:

(3) $$\vec{k_{a}}(r)=\vec{k_{b}}(r)+\vec{k}$$

where $\vec{k_{b}}(r)$ is the passband of the imaging system before spectrum shifting and $\vec{k_{a}}(r)$ is the passband after spectrum extension. Therefore, the cutoff frequency of the imaging system satisfies the following:

(4) $$\left||\vec{k_{b}}(r)|-|\vec{k}||\leq |\vec{k_{a}}(r)|\leq |\vec{k_{b}}(r)|+|\vec{k}\right|$$

**Figure 1:**
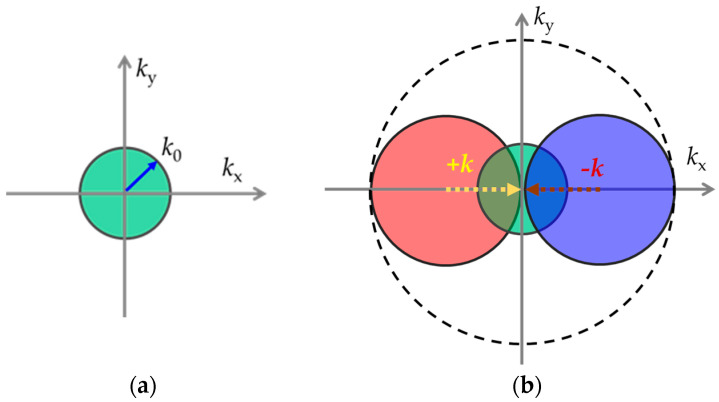
Fourier space OTF distribution. (**a**) The spectrum region that can be detected by traditional optical microscope; (**b**) the spectrum region that can be detected by light field illumination.

Figure 1a depicts the OTF function distribution under uniform light field illumination in the spectral domain. The diffraction limit of the optical imaging system restricts detection to spectral components below the cutoff frequency, specifically within the circular region. As shown in Figure 1b, under the action of the spatial frequency $\vec{k}$ of the illumination light field, two regions containing high-frequency information at a distance $\vec{k}$ from the spectrum space are moved to the central detectable area. Consequently, higher spatial frequencies allow for the detection of higher spectral components of the observed object, resulting in higher imaging resolution. Referring to Equation (2), it is evident that the surface plasmon light field excited at the same frequency possesses a higher spatial frequency, thereby enabling higher imaging resolution.

The essence of the optical diffraction limit is the inability of imaging processes to capture high-frequency details from the observed object. This limitation stems from light’s wave-like nature and the constraints of physical optics, preventing the imaging system from focusing light into an infinitely small point, as illustrated by the Airy disk (with a radius of approximately (0.61λ/NA) during the process of diffraction-limited focusing. Achieving super-resolution optical detection requires the strategic use of optical components to create a focal spot within the imaging system, thus capturing the object’s high-frequency components and, in turn, broadening the observable spectrum.

**Figure 2:**
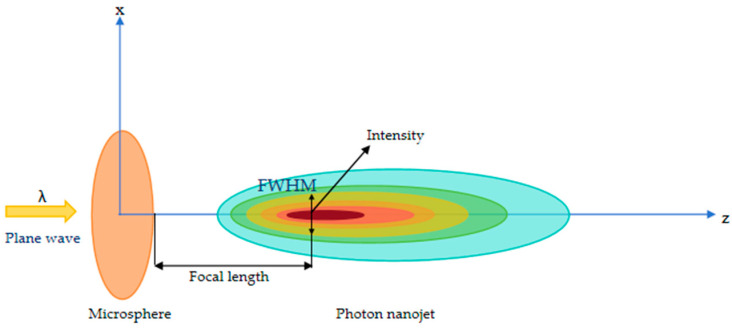
The PNJ of the nanosphere evanescent wave.

Figure 2 illustrates the schematic of the optical path, detailing the imaging process facilitated by a microsphere lens. The process begins with a wide beam of light emitted from the microscope, which the microsphere lens meticulously focuses and directs toward the object of interest. The light waves interact with the object upon reaching it, causing scattering. Subsequently, the scattered light waves are recaptured by the microsphere lens, which, together with the microscope, forms an image.

When a microsphere is irradiated with plane light, a photonic nanojet (PNJ) forms at its rear. The condition (*Z*_0_ ≪ *λ*) is satisfied when the PNJ is proximity close to the sample. Consequently, the incident light field *E*(*x,y,z*) = 1 on the sample surface contains both propagating and evanescent waves, suitable for use as the illumination field.

Following the application of the Fourier transform, the light field that has interacted with the imaging object is represented in the frequency domain as follows:

(5) $$E_{0}(c_{x},c_{y},0)=T(c_{x},c_{y})⊗E_{s}(c_{x},c_{y},0)$$

$T(c_{x},c_{y})$ is the transmission function. The *c_x_* and *c_y_* are the two components of the wave vector direction in the transmission function. After interacting with the imaging object $E_{0}(c_{x},c_{y},0)$, the light field propagates to the far-field detector for imaging purposes. The spatial spectrum on the detector plane can be described as follows:

(6) $$E_{0}(x,y,z_{\infty })=\int_{-\infty }^{+\infty }\int_{-\infty }^{+\infty }E_{0}(c_{x},c_{y},0)\exp[i(xc_{x}+yc_{y}+z_{\infty }c_{z})]dc_{x}dc_{y}$$

The transverse wave vector $c_{∥}=\sqrt{c_{x}^{2}+c_{y}^{2}}$ that can reach the detector must satisfy the following:

(7) $$c_{∥}\leq NA\cdot c_{0}$$

When the sub-wavelength structure (spectrum G=2π/δ) interacts with the incident light field (spectrum $c_{in}$), it can shift the wave vector of the incident light field to various diffraction orders, thus enabling the transformation of the light field’s spectrum.

(8) $$G-c_{in}\leq c_{out}\leq G+c_{in}$$

The spectrum *G* of the subwavelength fine structure of an object satisfies the following:

(9) $$G\leq c_{in}+c_{out}$$

The PNJ effect generates a highly localized light field upon the nanosphere’s exposure to incident plane light. The transverse length of the focal spot, known as the FWHM of the light intensity, is denoted by *H*. Consequently, the spectral properties of the incident light field on the object are as follows:

(10) $$c_{in}=\frac{\pi }{H}$$

After the incident evanescent wave interacts with the sample, it scatters off the observed object, returning it to below the cutoff frequency, and is subsequently detected by the charge-coupled device (CCD).

(11) $$c_{out}=c_{∥}$$

From Equations (7)–(11), it can be found that the spectrum *G* of the subwavelength fine structure of an object satisfies the following:

(12) $$G\leq NA\cdot c_{0}+\frac{\pi }{H}$$

Thus, the detectable size of the imaging object’s sub-wavelength structure must meet the following condition:

(13) $$\delta \geq \frac{\pi }{NA\cdot c_{0}+\pi /H}$$

Equations (3) and (13) indicate that the strong surface plasmon resonance (SPR) effect between nanospheres, when plane light is incident on microlenses, significantly increases the spatial frequency of the incident light spectrum, thereby capturing more high-frequency spectral components of the information. This increase in high-frequency components leads to a reduction in the length (*H*) of the generated PNJ. The reduction in PNJ length implies that the system’s minimum resolvable distance between two points decreases, significantly enhancing the imaging system’s resolution.

## 3. Simulation Model

**Figure 3:**
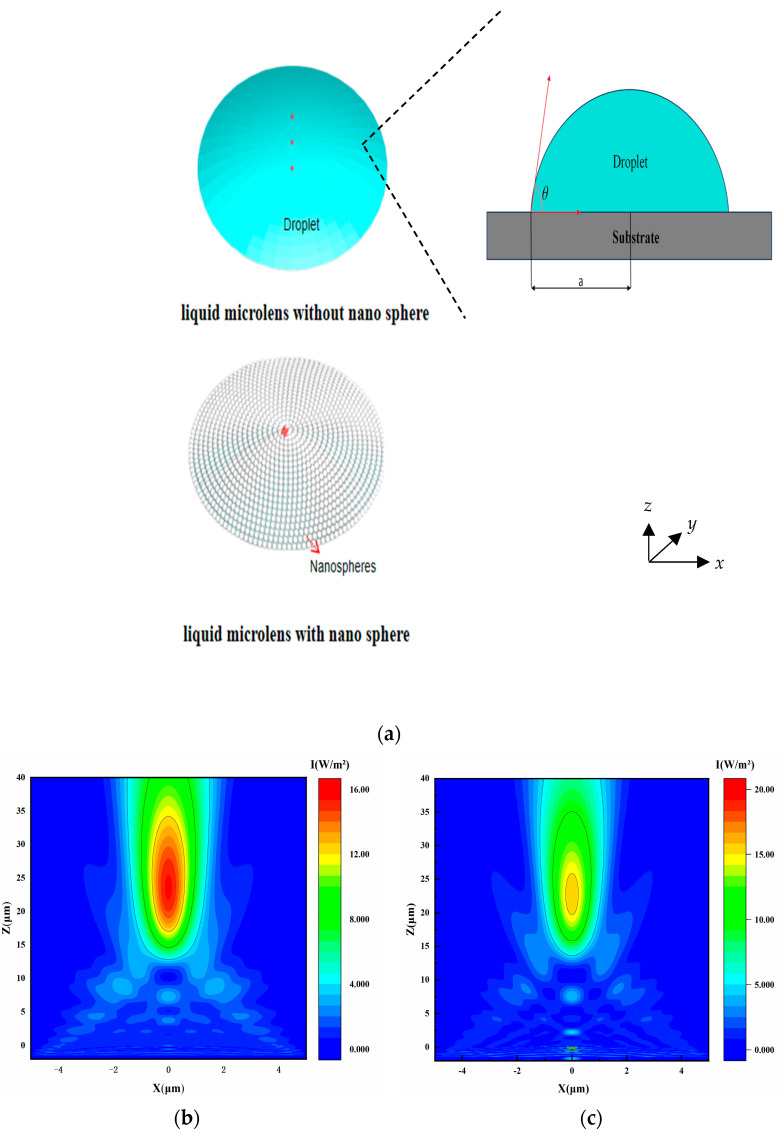

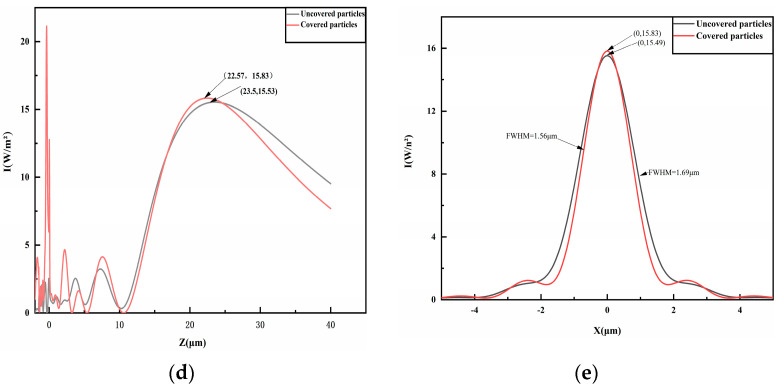
Model diagram of the microlens and FDTD software simulation results. (**a**) 3D view of a liquid microlens (*a* is the bottom radius, θ represents the initial contact angle). (**b**,**c**) The x–z plane light intensity distribution of the microlens without and with a microparticle, respectively. (**d**) Light intensity distribution in the z-direction for both microlenses at x = 0. (**e**) Light intensity distribution along the *x*-axis for the microlens without a microparticle and the microlens with a microparticle at z = 23.5 µm and z = 22.57 µm.

The metasurface encased liquid microlens is designed to achieve super-resolution microscopy by utilizing the plasmonic properties of Au@TiO_2_, Core-Shell nanospheres, allowing manipulation of the optical frequency spectrum. These Au@TiO_2_, Core-Shell nanospheres, configured uniformly and densely as a metasurface, surround the liquid microlens. To accurately examine the encapsulated structure, Figure 3a displays schematic representations of the liquid microlens in its bare state at the top and enveloped by the metasurface at the bottom, respectively. 

The simulations are conducted using the FDTD method (Ansys Lumerical 2020 R2 Finite Difference IDE, Lumerical, Vancouver, BC, Canada) on a laptop with an Intel Core i7-14700Hx CPU and 16 GB RAM. The spatial mesh is set to 30 nm for all dimensions (∆x = ∆y = ∆z = 30 nm). The light source is configured as a plane wave along the *Z*-axis, with an incident light wavelength *λ* ranging from 400 to 700 nm and an intensity of 1 W/m^2^. To ensure result convergence, the time step is set to 0.076263 fs, and the simulation duration is 1000 fs. Two droplets (Glycerol), identical in bottom diameter (a = 9.6 µm), initial contact angle (θ = 20°), and refractive index, are modeled as liquid microlenses: one is coated by Au@TiO_2_, Core-Shell nanospheres, and the other is not. The radius of the nanosphere was set to 50 nm. The gap between adjacent nanospheres is set to 100 nm to ensure close contact. The simulation boundary employs the Perfectly Matched Layer (PML) absorbing boundary condition.

Figure 3b,c shows the PNJs generated by microlenses focusing light waves under the same conditions. The calculated light intensity distributions along the central x and y axes of these PNJs are shown in Figure 3d,e. These figures indicate that the particle-free microlens has a maximum light intensity of 15.49 W/m^2^, while the microlens with particles achieves a higher intensity of 15.83 W/m^2^. Consequently, the particle-coated microlens exhibits enhanced focusing capabilities, which are beneficial for super-resolution imaging. This improvement is mainly due to Au@TiO_2_, Core-Shell’s high refractive index. Applying Au@TiO_2_, Core-Shell as a coating on the liquid microlens enhances light absorption and manipulates the light propagation path effectively. Additionally, the uniform distribution of Au@TiO_2_, Core-Shell particles across the lens surface, resembling a thin film, promotes uniform light diffusion and scattering, further enhancing the lens’s focusing capabilities.

The FWHM of a microlens’s focus corresponds to the Airy disk produced by the diffraction of parallel light through the lens, a crucial parameter for assessing the lens resolution. It is directly related to the light spot size, numerical aperture, and diffraction limit. The formula FWHM ≈ *λ*/(2*NA*) indicates that a smaller FWHM results in higher lens resolution, as it suggests a smaller focal spot and the ability to resolve finer details. Figure 3e shows that the FWHM for the particle-free microlens is 1.69 µm, and, for the microlens with particles, it is 1.56 µm, highlighting the advantage of the particle-coated microlens for imaging magnification and observing super-resolution details.

## 4. Results

To investigate how the initial contact angle of the droplet, the nanosphere radius, and the droplet bottom diameter affect super-resolution imaging, we simulate their PNJ effects.

### 4.1. Initial Contact Angle of Droplets

**Figure 4:**
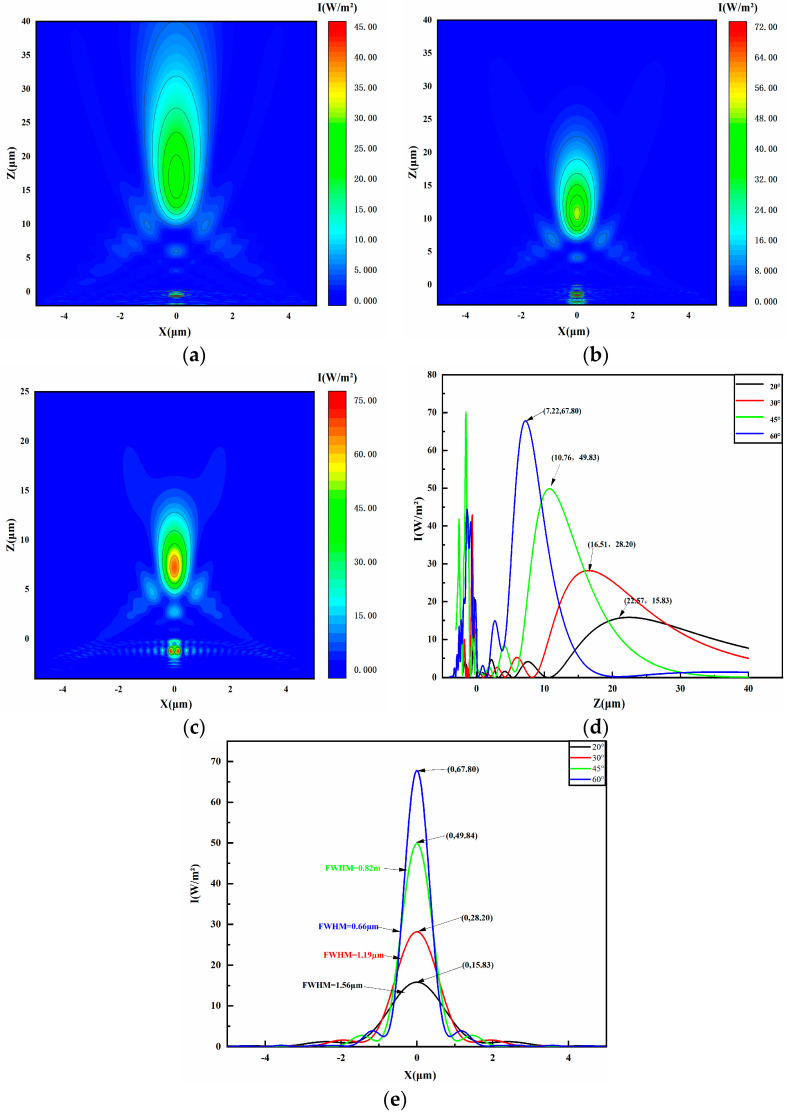
Visualized electric field intensity distributions calculated by FDTD by varying the initial contact angle of the metasurface-coated liquid microlenses. (**a**–**c**) The light intensity distribution for initial contact angles of 30°, 45°, and 60°, respectively. (**d**) The light intensity distribution of the microlens along the *z*-axis at x = 0. Figure (**e**) illustrates the light intensity distribution and full width at half maximum (FWHM) along the *x*-axis for microlenses with initial contact angles of 20°, 30°, 45°, and 60° at z = 22.57 µm, z = 16.51 µm, z = 10.76 µm, and z = 7.22 µm.

Research findings show that the characteristics of PNJs significantly change with variations in the initial contact angle of microlenses. To study the effect of the initial contact angle on PNJs, simulations were conducted with control groups having initial contact angles of 20°, 30°, 45°, and 60°. The simulated light intensity results are presented in Figure 3c and Figure 4a–c. Observations indicate that increasing the initial contact angle of the microlens decreases both the focal and effective lengths of the PNJs. The variations in light intensity and FWHM, derived from the simulation data in Figure 3c and Figure 4a–c, are graphed in Figure 4d,e. For microlenses with initial contact angles of 20°, 30°, 45°, and 60°, the light intensities are 15.83 W/m^2^, 28.20 W/m^2^, 49.84 W/m^2^, and 67.80 W/m^2^, respectively, while the FWHM values are 1.56 µm, 1.19 µm, 0.82 µm, and 0.66 µm, respectively. Data indicate that, as the initial contact angle increases, the light intensity of PNJ in the field distribution produced by the liquid microlens gradually increases, while FWHM shows a decreasing trend. With an increasing initial contact angle, the near-field coupling of the liquid microlens improves, allowing for more efficient energy transfer of incident light into the microlens. Consequently, this affects the focal length of the PNJ and strengthens the light’s focus, resulting in increased PNJ intensity. Additionally, as the initial contact angle grows, the evanescent waves excited by the incident light on the microlens surface align better with the waveguide mode’s propagation constant, enhancing coupling efficiency. This leads to modifications in the incident light’s propagation within the waveguide, changing the light waves’ focusing properties and reducing the PNJ’s focal length. Ultimately, an increase in the initial contact angle modifies the microlens’s curvature radius and adjusts the incident light’s angle, optimizing plasmonic resonance among nanoparticles on the microlens surface and boosting the local electric field, thereby further enhancing PNJ intensity.

### 4.2. Radius of Nanospheres

**Figure 5:**
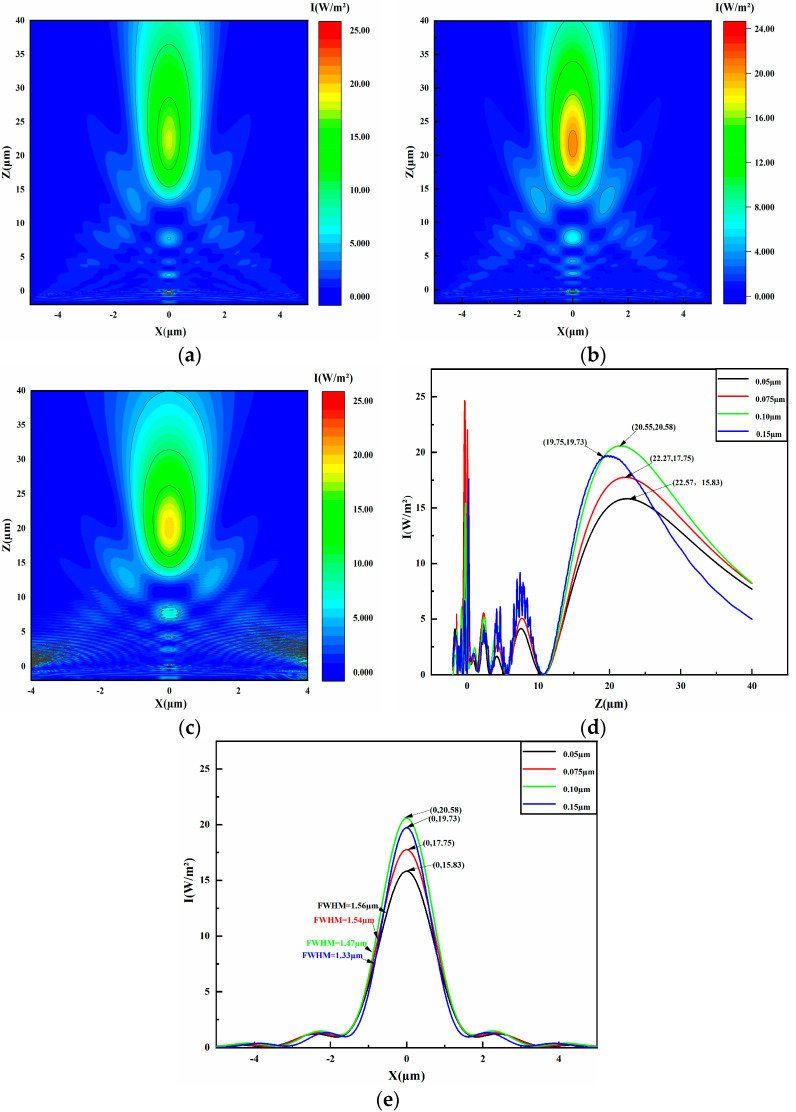
The visualizations of light intensity distributions, obtained through FDTD computations, are presented for nanospheres with diameters of 75 nm, 100 nm, and 150 nm. Subfigures (**a**–**c**) depict the light intensity distributions for nanospheres of 75 nm, 100 nm, and 150 nm, respectively. Subfigure (**d**) delineates the light intensity profile of the microlens along the *z*-axis at x = 0. Subfigure (**e**) details the light intensity distribution and full width at half maximum (FWHM) along the *x*-axis for microlenses with nanosphere diameters of 75 nm, 100 nm, and 150 nm at the z-coordinates of 22.27 µm, 20.55 µm, and 19.75 µm.

The size of nanospheres substantially affects the intensity and focal length of PNJs. In the Rayleigh scattering regime, where nanosphere radii are much smaller than the incident light wavelength, PNJ formation is minimal. However, in the Mie scattering regime, where particle diameters exceed the incident light wavelength, PNJs are effectively generated. To understand how nanosphere size impacts PNJ generation on liquid microlenses, we conducted simulation experiments with radii of 50 nm, 75 nm, 100 nm, and 150 nm. The resulting light intensity distributions are shown in Figure 3c and Figure 5a–c. Increasing nanosphere size leads to a gradual decrease in PNJ length and focal length. The variations in light intensity and FWHM, derived from these simulations, are plotted in Figure 5d,e. For microlenses with nanosphere radii of 50 nm, 75 nm, 100 nm, and 150 nm, the light intensities are 15.83 W/m^2^, 17.75 W/m^2^, 20.55 W/m^2^, and 19.73 W/m^2^, respectively, and the FWHM values are 1.56 µm, 1.54 µm,1.47 µm, and 1.33 µm, respectively. Data indicate that the light intensity of PNJs produced by the microlens increases and then decreases with the increase in microsphere size, reaching a maximum value when the microsphere size is 100 nanometers, while FWHM shows a decreasing trend. The main reason for this phenomenon is that, at smaller microsphere sizes, the electric dipole resonance plays a dominant role; however, when the size increases to 100 nanometers, the electric quadrupole resonance in the short-wavelength region becomes significant and surpasses the electric dipole resonance. This transition leads to an increase in PNJ light intensity, peaking at a microsphere size of 100 nanometers, as the surface plasmon resonance between nanoparticles on the microlens surface is most conducive to beam focusing. With the increase in microsphere size, the focusing ability of the microlens is enhanced, causing the beam to become more concentrated, thereby reducing the FWHM. Additionally, the evanescent waves generated on the spherical surface of the microlens are effectively coupled and transmitted, converting evanescent waves into propagating light waves, which shortens the focal length of the microlens and enhances the light-focusing effect. However, when the microsphere size exceeds 100 nanometers, the length of PNJ increases, leading to changes in the energy distribution at the focus and thus causing a decrease in light intensity.

### 4.3. Diameter of Droplet Bottom

**Figure 6:**
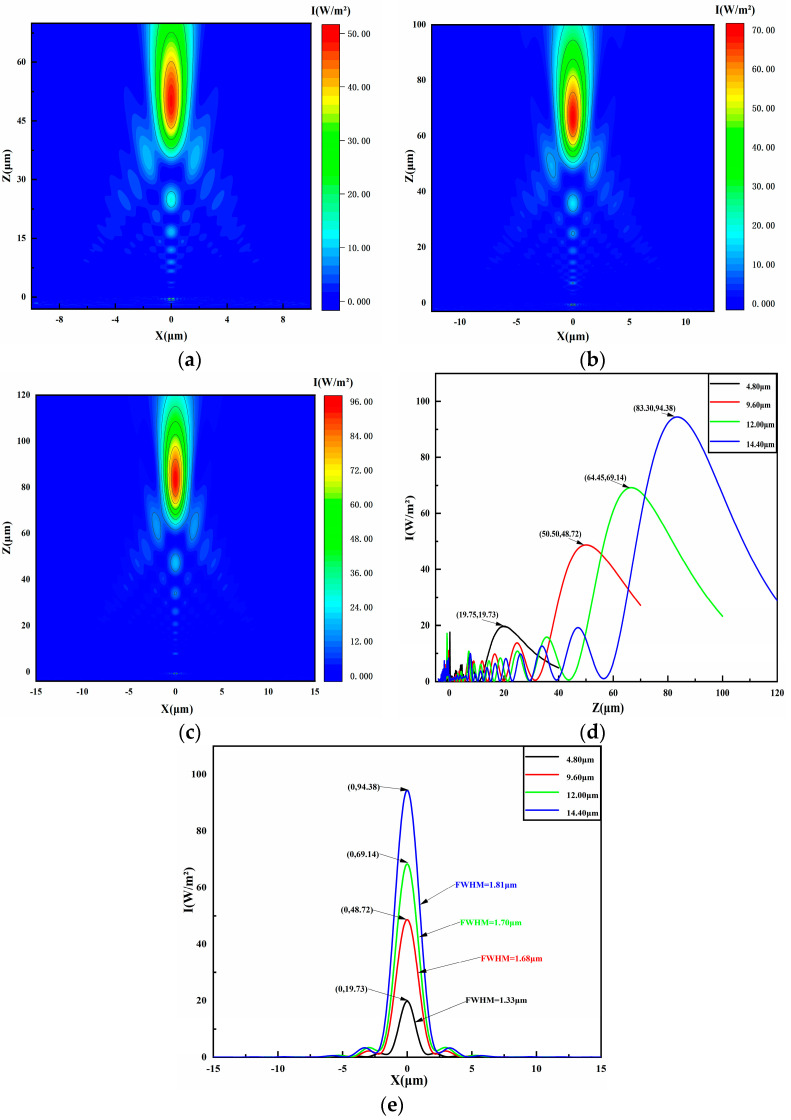
Visualized electric field intensity distributions calculated by FDTD by varying the droplet bottom diameter. (**a**–**c**) The light intensity distribution of metasurface-coated liquid microlenses with base radii of 9.60 µm, 12.00 µm, and 14.40 µm, respectively. (**d**) The light intensity distribution of the microlens along the *z*-axis at x = 0. (**e**) The light intensity distribution and full width at half maximum (FWHM) along the *x*-axis for microlenses with base radii of 4.80 µm, 9.60 µm, 12.00 µm, and 14.40 µm at z-coordinates of 19.75 µm, 50.50 µm, 66.45 µm, and 88.30 µm.

To determine how microlens base radius affects PNJs performance, we conducted simulations with base radii of 4.80 µm, 9.60 µm, 12.00 µm, and 14.40 µm, maintaining a constant nanosphere radius at 150 nm, incident light wavelength range of 400–700 nm, and an initial contact angle of 20°. The simulated light intensity results are presented in Figure 5c and Figure 6a–c. Observations show that increasing the microlens base diameter leads to increased focal and effective lengths of the PNJs. The variations in light intensity and FWHM, derived from the simulation data in Figure 5c and Figure 6a–c, are graphed in Figure 6d,e. For microlenses with base radii of 4.80 µm, 9.60 µm, 12.00 µm, and 14.40 µm, the light intensities are 15.83 W/m^2^, 48.72 W/m^2^, 69.14 W/m^2^, and 94.38 W/m^2^, respectively, and the FWHM values are 1.56 µm, 1.68 µm, 1.70 µm, and 1.81 µm. The results indicate that increasing the microlens base radius leads to higher light intensity and wider FWHM of the PNJs. This trend is primarily attributed to the increased interaction area between the incident light and the liquid microlens due to the larger base radius, which captures more photons and enhances plasmonic effects in more nanospheres, significantly boosting local electric field intensity and microlens focusing, thus increasing light intensity. Additionally, an increase in the base radius alters the microlens’s curvature, influencing the light’s propagation and increasing the PNJs’ focal length, which, in turn, increases the microlens’s FWHM.

## 5. Conclusions

This paper introduces a successfully designed compound structure liquid microlens that exhibits superior FWHM and light intensity compared to single-structure microlenses. Furthermore, we have conducted an in-depth exploration of the effects of the microlens’s initial contact angle, nanosphere size, and base radius on the characteristics of PNJs. Through a series of simulation experiments, we found that an increase in the initial contact angle leads to a reduction in the focal length and effective length of PNJs, an increase in light intensity, and a narrowing of FWHM. Similarly, an increase in nanosphere size also affects the length and focal distance of PNJs, as well as light intensity and FWHM. Additionally, an increase in the base radius of the microlens extends the focal length and alters the curvature radius of the microlens, resulting in an increase in both light intensity and FWHM. These findings indicate that, by precisely controlling the design parameters of the microlens, we can optimize its potential in super-resolution imaging applications, providing new avenues for future optical imaging technologies.

## Data Availability

The original contributions presented in this study are included in the article. Further inquiries can be directed to the corresponding author.
